# A phase II study of thalidomide and temozolomide in patients with brain metastases from malignant melanoma: lymphopenia correlates with response

**DOI:** 10.3332/ecancer.2008.91

**Published:** 2008-08-15

**Authors:** LW Vestermark, E Holtved, R Dahlrot, MK Brimnes, IM Svane, L Bastholt

**Affiliations:** 1 Department of Oncology, Odense University Hospital, DK-5000 Odense C, Denmark; 2 Center for Cancer Immune Therapy, Department of Hematology, Herlev Hospital, Denmark; 3 Department of Oncology, Herlev Hospital, Denmark

## Abstract

**Background:**

Central nervous system (CNS) metastases develop in nearly half of patients with advanced melanoma and in 15–20% CNS is the first site of relapse. Median overall survival is short, ranging from two to four months, and one-year survival rate is only 10–15%. THA has been shown to have both anti-angiogenetic and immuno-modulating effects. TMZ is an oral alkylating agent with an excellent oral bioavailability and it is highly lipophillic with an ability to penetrate the blood–brain barrier. TMZ and THA in combination were tested in patients with brain metastases from malignant melanoma.

**Methods:**

Between June 2004 and February 2007 patients with measurable metastatic melanoma in progression and PS ≤ 1 received TMZ in a dose of 150 mg/m^2^ qd for seven days, followed by seven days off therapy and THA in 200 mg qd, both orally administered. Concomitant treatment with steroids was allowed. PBMCs were collected from the last 14 consecutive patients for evaluation of immune parameters.

**Results:**

Forty screened patients were eligible and evaluable for response, and 39 were evaluable for toxicity. 25 patients had asymptomatic and 15 symptomatic brain metastases. The toxicity was primarily grade 1–2 with no grade 4 or treatment-related deaths. Four patients had thromboembolic events grade 3. One patient obtained a CR and five a PR in the CNS, while two had CR and four had PR outside CNS. Overall response rate was 17.5%. We found a significant positive correlation between lymphopenia and objective response.

**Conclusions:**

The combination treatment was well tolerated but with more frequent thromboembolic events compared to single drug TMZ or THA. The treatment demonstrated activity in CNS as well as outside CNS. The correlation between lymphopenia and objective response needs further investigation.

## Introduction

Malignant melanoma (MM) in the advanced stage has a poor prognosis. The disease is notorious for its propensity to metastasize and for its poor response to current medical therapeutic regimens, with median survival times in most studies ranging from six to nine months and five-year survival rate of less than 5% [1]. Furthermore, melanoma is the third most common cause of metastases in the central nervous system (CNS) after carcinomas of the lung and breast and develops in nearly 50% of patients with metastatic melanoma. CNS metastases is the first failure site in approximately 40% among the 8–10% of patients with an initial complete response to high-dose IL-2-based therapy [2]. Median survival time in these patients is short, ranging from two to four months, and one-year survival rate is 10–15% [3].

Once patients develop brain metastases, treatment is palliative. Surgery and stereotactic radiotherapy can produce effective palliation in patients with a few CNS lesions [4]. In case of multiple or inoperable CNS metastases, whole brain radiotherapy is considered standard of care. Temozolomide (TMZ) is an oral, alkylating agent with a high oral bioavailability. It is highly lipophilic with an ability to penetrate the blood–brain barrier, and it has demonstrated activity in the treatment of brain metastases from MM [5]. The schedule for treatment with TMZ is of utmost importance. Compared to the standard dose of 150–200 mg/m^2^ five days every 28 days, extended dose permits a 2.1-fold greater drug exposure over four weeks. Different schedules have been tried [6,7]. A lower dose per day but given continuously over a period of 6–8 weeks followed by a two-week rest has led to significant toxicity, with lymphopenia in up to 75% of the patients treated [8,9]. An increased depletion of the DNA repair enzyme 0(6)-alkylguanin-DNA alkyltransferase (AGAT) has been observed with the extended dosing, which may lead to enhanced anti-tumour activity. In patients treated with TMZ, the activity of the enzyme was measured in mononuclear cells in the blood [7]. Thalidomide (THA) was introduced in Europe and Canada in the late 1950s as a sedative and for the treatment of morning sickness in pregnant women. The drug was withdrawn from the market in the early 1960s because reports of teratogenecity associated with its use emerged [10]. D’Amato *et al* were the first to show the inhibitory activity of THA on angiogenesis in a rabbit model of corneal neovascularization induced by basic fibroblast growth factor (bFGF) and/or vascular endothelial growth factor (VEGF) [11]. Furthermore, THA inhibits the inflammatory response by decreasing cyclo-oxygenase-2 activity and by suppressing the synthesis of tumour necrosis factor-a (TNF-a) [12] by inducing TNF-a mRNA degradation [13]. THA enhances the production of IL-2 and IL-2 receptors, modulating the immune system to induce anti-cancer activity. The function of other cytokines, such as IL-6 and IL-12, are inhibited by THA, and it also modulates the expression of cell adhesion molecules [14–16]. THA has demonstrated clinical activity against a range of solid tumours, including melanoma [17]. We found in a phase-II trial that THA as single agent has some extra-cranial activity in patients with brain metastases from MM but no objective response was seen in the brain [18]. Hwu *et al* reasoned that additional benefit might be gained by combining TMZ with THA and that the combination of continuous daily oral TMZ and THA appears to be a promising oral regimen for the treatment of MM, particularly in patients with brain metastases [19]. With the intention of optimizing this regimen we have conducted a phase-II trial with TMZ using extended dose, 150 mg/m^2^ qd for seven days, followed by seven days off therapy and THA 200 mg qd.

## Materials and methods

### Patients

All patients included in the study had brain metastases in progression not amenable for surgery or stereotactic radiotherapy and due to limited symptoms whole brain radiotherapy was not indicated. WHO performance status (PS) ≤ 0.1. Further inclusion criteria were: measurable disease in the brain, defined as ≥ 1.0 cm in diameter on spiral CT or MRI. Concomitant steroids were allowed for patients with clinical evidence of brain metastases. Lesions within a previous radiation field had to have shown recent disease progression to be considered evaluable. Previous treatment with IL-2, other biologic therapy or radiotherapy must have been completed ≥ 4 weeks prior to study enrolment. Eligible patients also met the following criteria: WBC ≥ 3.0 × 10^9^/l, ANC ≥ 1.5 × 10^9^/l, platelets ≥ 100 × 10^9^/l, serum alanine aminotransferase and alkaline phosphatase levels < 2.5 UNL and bilirubin and creatinine ≤ 1.5 UNL. All patients were obliged to comply with Pharmion’s Risk Management Programme, conducted according to the System for Thalidomide Education and Prescribing Safety (STEPS) guidelines [20]. Female patients were required to have negative urine pregnancy test, within 24 hours prior to THA therapy. Any women of child-bearing potential had to use adequate contraceptive methods during the study and for 28 days after the last THA dose. Men were required to use condoms in connection with any sexual activity. Pregnant or nursing women were excluded. All patients provided written informed consent.

### Treatment and patient evaluation

Starting dose of THA was 100 mg qd orally, taken in the evening to reduce drug-related daytime somnolence. The dose was escalated after one week to a maximum of 200 mg qd given continuously. Concomitantly, patients received treatment with TMZ 150 mg/m^2^ qd for seven days, followed by seven days off therapy. Treatment was continued until unacceptable toxicity, patient refusal or disease progression.

Toxicity was assessed according to Common Toxicity Criteria (CTC v3.0) [21]. The dose of THA was interrupted in cases of myelotoxicity, constipation or somnolence grade 3–4 and could be resumed if toxicity was reduced to grade 1. In cases of treatment interruption due to THA related myelotoxicity or constipation, somnolence (≥ grade 2) or neuropathy (> grade 2), treatment could be restarted with 50% dose reduction. No dose re-escalation was performed. In cases of grade 4, neuropathy treatment was stopped.

Dose modification guidelines for TMZ allowed for interruption of treatment in the event of any grade 3–4 toxicity, until toxicity resolved to ≤ grade 1 or baseline level. Subsequently, TMZ would be restarted at a dose of 112.5 mg/m^2^/d. With subsequent toxicity > grade 2 doses would be reduced to 75 mg/m^2^/d, which was the lowest allowed dose. Re-escalation of the dose was not allowed.

One treatment cycle was defined as a four-week period. Clinical evaluation was performed after the initial two and four weeks in order to perform proper dose correction. Thereafter, patient clinical evaluation was performed at four-week intervals to assess toxicity and clinical benefit. During each visit, a complete blood count was taken, combined with evaluation of blood pressure, weight and performance status.

Response to treatment was assessed after three cycles according to RECIST [22], by physical examination and CT or MRI of all metastatic sites. In case of disease progression prior to first evaluation, treatment was stopped and the patient was designated as having progressive disease (PD). In case of stable disease (SD) or an objective response, partial response (PR) or complete response (CR), treatment was continued.

### Identification of lymphopenia

The absolute lymphocyte count (ALC) was determined together with the complete blood count during each visit. Lymphopenia was defined as ALC < 0,8 × 10^9^/l corresponding to CTC grade 2. Severe lymphopenia was considered to be ALC < 0.5 × 10^9^/l (≥ grade 3 lymphopenia). Initially, prophylactic antibiotics were not administered for lymphopenia, but following cases of opportunistic infections occurring among the first 15 patients enrolled in the study. We started to administer prophylactic antibiotics (Sulfamethoxazol/Trimetoprim two tablets three times a week) to patients with grade 2 lymphopenia and fever > 38º and to patients with ≥ grade 3 lymphopenia with or without fever.

### Analysis of T-cell populations

Three peripheral blood samples were obtained from the last 12 patients enrolled in the study. One before treatment with THA and TMZ (pre-sample), one after treatment day 14 and one on day 28. Mononuclear cells (MNC) were purified using leucosep^®^ tubes (Greiner Bio-One GmbH, Germany). Red blood cells were lysed and MNCs were cryopreserved in foetal bovine serum (FBS) (Gibco, Invitrogen, Carlsbad, CA, USA) containing 5% DMSO until use. For staining of CD4^+^CD25^+^FOXP3^+^ T_reg_ cells the PE anti-human FOXP3 staining kit from eBioscience was used (eBioscience, San Diego, CA, USA). In brief, cells were stained with FITC anti-human CD127, PE anti-human FOXP3, PerCP anti-human CD4, APC anti-human CD25 and the relevant isotype controls as recommended by the manufacturer. The cells analysed on a FACSdiva (Becton Dickinson). The collection was stopped after acquiring 200,000 events, and data were analysed using FACSdiva software (Becton Dickinson). For defining the population of T_reg_ cells, the cells were first gated on CD4^+^ lymphocytes then on CD4^+^ and CD25^high^. On the pre-sample, the level of CD4^+^CD25^high^ out of CD4^+^ T cells was fixed at 2%, and the following two samples (day 14 and day 28) were analysed on the same day using the same gates. To confirm that the cells within the gate were T_reg_ cells, we evaluated the FOXP3 expression and found the FOXP3 expression to be between 80–95%. When evaluating the level of CD4^+^CD127^+^CD25^neg-int^ T cells, the cells were gated on CD4^+^ lymphocytes then on CD127^+^ and CD25^neg-int^.

## Statistical analysis

This single-institution phase-II study was approved by the Ethics Committee of Funen County. The primary objective of the study was to determine response rate of the study treatment according to RECIST, whereas estimation of time to progression, overall survival and evaluation of tolerability of the regimen were secondary objectives. Patients were accrued to this study using a two-stage Simon’s design [23] in order to improve the probability of observing the true response rate. Thus, if there were three or more responders among the first 21 evaluable patients, the accrual would be extended to a total of 40 evaluable patients with five patients per responder with an estimated response rate of 25%. Descriptive statistics were used to describe baseline patient characteristics, the cerebral and extra-cerebral objective response rate and toxicity. Time to progression and death were estimated using the Kaplan-Meier method, with overall survival measured from study entry until death from any cause. The T-cell data were analysed using a student *t* test.

## Results

### Patient characteristics

A total of 43 patients were enrolled in the study between June 2005 and February 2007. Three patients did not meet protocol eligibility requirements and were excluded from all analyses. Baseline patient and disease characteristics for 40 eligible patients (21 men, 19 women) are shown in Table 1. Thirty-nine patients were evaluable for toxicity as one patient died shortly after the start of treatment. Median age of the patients was 55 years (range: 29–76 years), 16 patients were in WHO PS: 0 and 24 in PS: 1. All patients had non-resectable brain metastases in progression: 15 had minor neurologic symptoms and were treated with concomitant steroids. The remaining 25 patients had asymptomatic brain metastases all found during screening procedures prior to planned IL-2-based therapy. Among 40 patients, 34 had known primary cutaneous melanoma, six had unknown primary melanoma. Thirty-nine patients had concomitant extensive extra-cranial disease with 15% having liver metastases and 57% having lung metastases. Before study entry, four patients had received whole-brain radiotherapy, one had received stereotactic radiotherapy and three had undergone surgery for brain metastases. Seven patients had received prior immunotherapy and none prior cytotoxic therapy. The patients completed a median of three cycles of therapy (range: 1–10 cycles). Eight patients received only one cycle because of early clinical disease progression. Twenty-seven patients completed three cycles and were evaluated for efficacy.

### Analysis of lymphopenia

All 40 patients were analysed for lymphopenia. Eight patients experienced no lymphopenia during treatment; five patients experienced grade 1; eight patients grade 2; 16 patients grade 3; and eventually three patients experienced grade 4 lymphopenia (< 0.2 × 10^9^/l). The observed lymphopenia rate was 68%.

### Analysis of T-cell populations

A total of 12 patients were analysed for the presence of CD4^+^CD25^+^FOXP3^+^ T_reg_ cells. None of the patients included in the analysis had objective response during treatment with THA and TMZ (eight patients had PD and four patients had SD). The percentage of T_reg_ cells out of CD4 T cells was increased after treatment with TMZ and THA (p=0.001) (Figure 1A), while the total number of T_reg_ cells was unchanged after treatment (p=0.8) (Figure 1B). The percentage of CD4 T cells out of lymphocytes (p=0.03) as well as the total number of CD4 T cells (p=0.04) decreased during treatment (Figure 2A and B). Furthermore, a decreased percentage of CD4^+^CD127^+^CD25^neg-int^ T cells out of CD4 T cells (p=0.0001) along with a tendency to a decreased number of total CD4^+^CD127^+^CD25^neg-int^ T-cells were observed (Figure 3). However, the latter did not reach statistical significance (p=0.2).

### Toxicity

The reported non-haematologic adverse events are summarized in Table 2. The toxicity was primarily grade 1–2 with fatique, constipation and mouth dryness. Four had thromboembolic events (TEE) all grade 3, which did not result in discontinuation of therapy. These patients were treated with anti-coagulants. Except for one patient with pancytopenia grade 3, no significant neutropenia or thrombocytopenia and no grade 4 or treatment-related deaths was observed. Thirteen patients were treated with antibiotics because of lymphopenia. Among these, three patients had opportunistic infections or other infections indicative of T-cell dysfunction with one patient having systemic infection with Candida Albicans in the lungs and two patients with Legionella. During the study, only two patients discontinued study treatment because of toxicity. One patient discontinued because of grade 3 nausea and vomiting, possibly related to the study drug and one patient because of grade 3 haematologic toxicity. Disease progression was the most common reason for treatment discontinuation (62% of cases).

## Response

Objective response rates according to RECIST in the brain and outside CNS are shown in Table 3. Among the 40 patients, one patient obtained a CR in the brain, five a PR and ten a SD. Outside CNS, two patients obtained a CR, four a PR and ten a SD. One patient obtained a CR overall, while six patients had PR, leading to an overall response rate of 17.5%. Unfortunately, planned confirmation of response according to RECIST was not a part of our protocol; therefore, only four out of seven responses were confirmed after three months. A further eight patients had SD in both CNS and outside CNS, leading to a clinical benefit rate of 37.5%. The three patients referred to as non-evaluable for response outside the CNS were all patients with metastases in the brain as the only metastatic site. Among the seven patients who had an overall objective response, three were treated with concomitant steroids, four had symptomatic and three had asymptomatic brain metastases.

**Figure 4:**
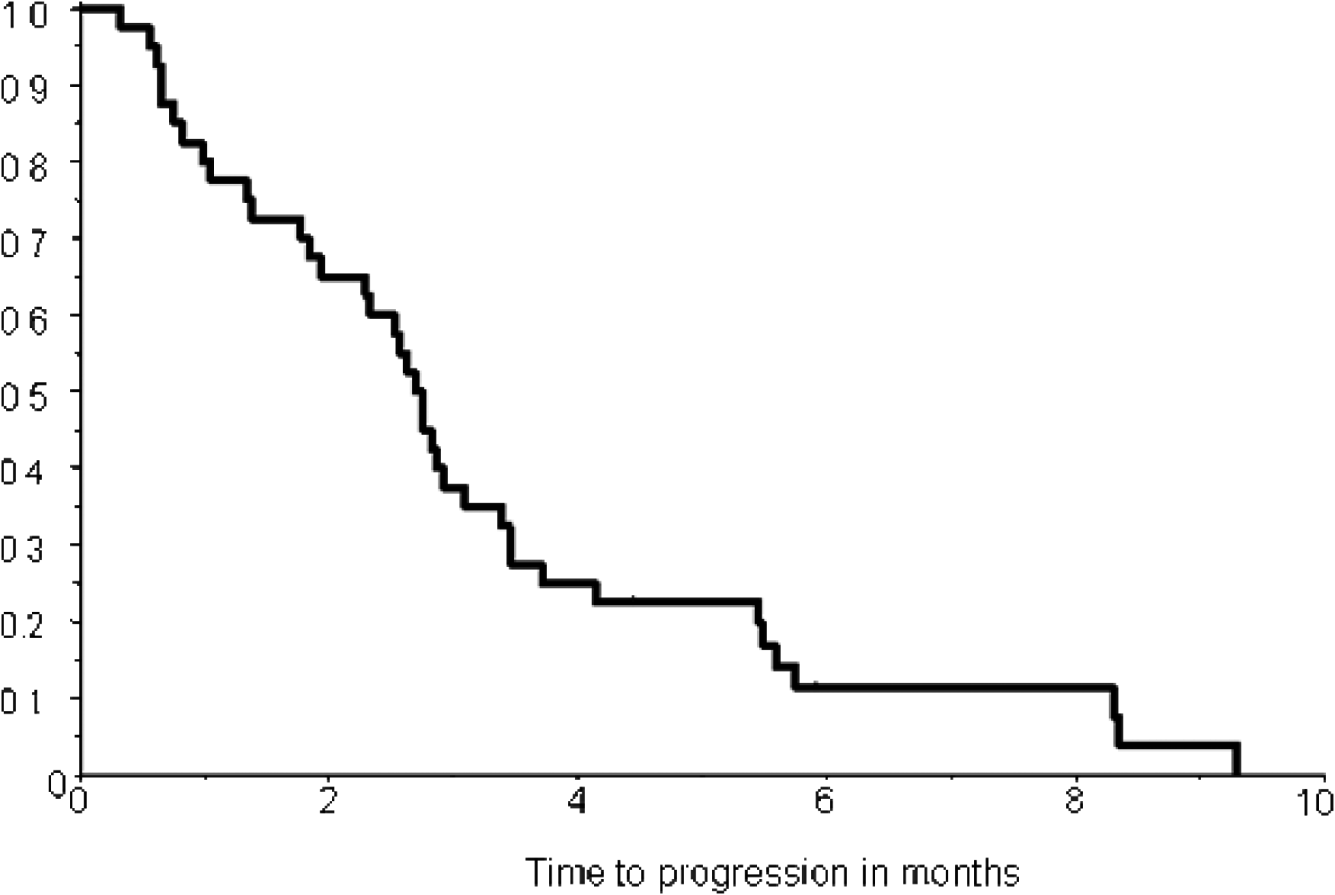
Kaplan-Meier estimate of time to progression in 40 patients. Median time to progression is 2.7 months (95% conf lim: 0.3–9.3 mths)

**Figure 5:**
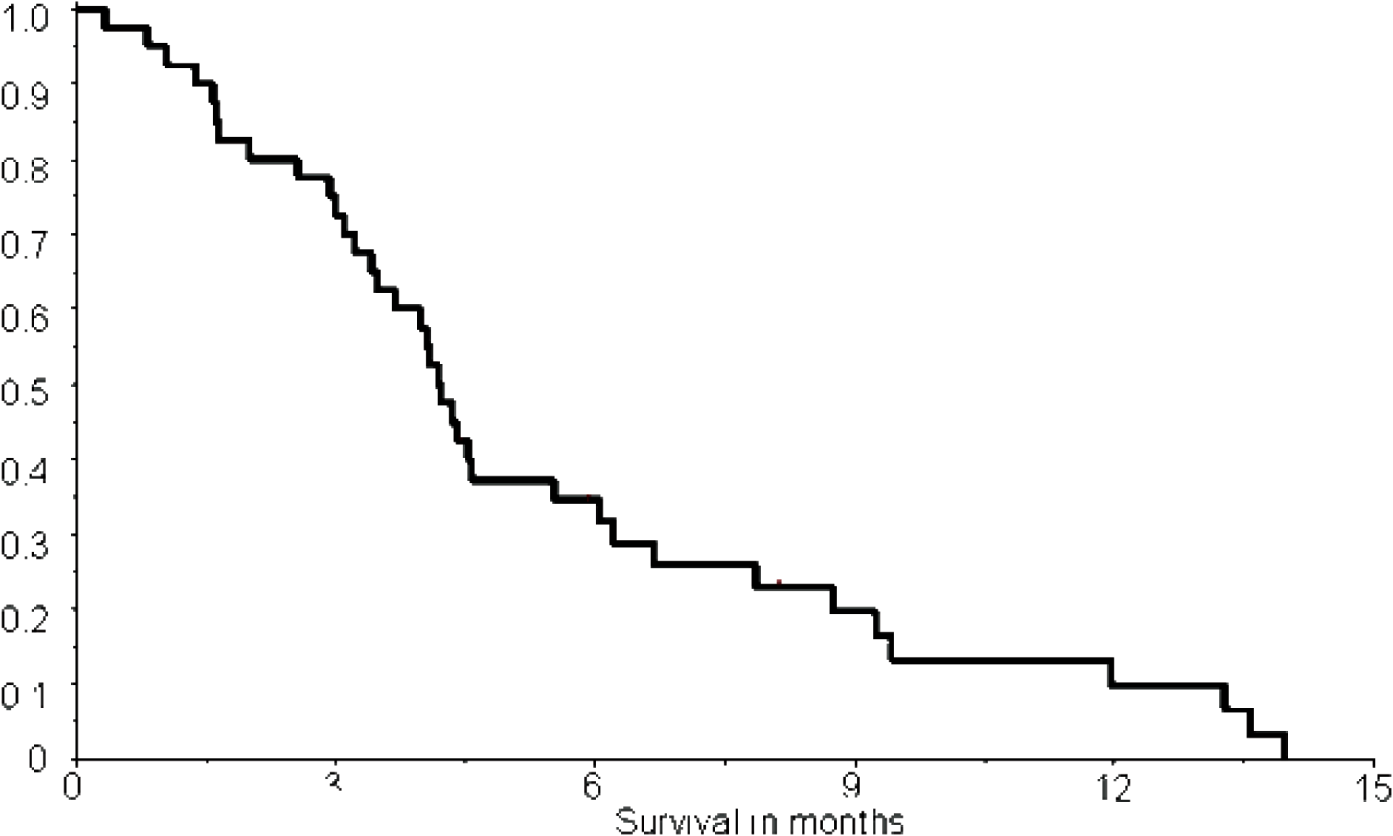
Kaplan-Meier estimate of overall survival in 40 patients. Median survival time is 4.2 months (95% conf lim: 0.8–14.0 months)

The correlation between lymphocyte count and response to treatment is shown in Table 4. Patients with SD have been categorized together with patients obtaining an objective response. We find a statistically significant correlation between efficacy and lymphopenia (Fishers exact test, p= 0.01). The Kaplan-Meier estimate of time to progression is shown in Figure 4. Median time to progression was 2.7 months (95% conf lim: 0.3–9.3 months). Kaplan-Meier estimate of overall survival is shown in Figure 5. The median survival time was 4.2 months (95% conf lim: 0.8–14.0 months). No difference in survival comparing patients with symptomatic (*n*=15) and asymptomatic (*n*=25) brain metastases was found (data not shown).

## Discussion

In this phase II trial, we have investigated the combination of THA and a dose-intensified schedule of TMZ 150 mg/m^2^ seven days on/seven days off, in the treatment of malignant melanoma with brain metastases. In our study, we observed a response rate of 17.5% and a clinical benefit rate (CR + PR + SD) of 37.5%. The response rate using the classical schedule of TMZ single drug leads to a response rate of 6–9% [24,25], with further 10% obtaining SD. These two studies were the first to demonstrate any anti-tumour activity of TMZ in brain metastases of melanoma patients. A phase-II study by Schadendorf *et al* [26] using dose-intensified bi-weekly TMZ did not lead to significant response in brain metastases or extra-cerebral metastatic lesions (under 5%) [26]. A small study with extended dose of TMZ (six weeks, low dose) combined with THA showed a response rate of 12% and a clinical benefit of up to 31% in 26 patients [19,27]. Our study is the first phase-II study published combining the cyclic (seven days on/seven days off) regimen of TMZ and THA for patients with malignant melanoma suffering from brain metastases, showing a meaningful response rate.

Su *et al* were the first to report lymphopenia and opportunistic infections as an unexpected toxicity of extended-dose regimens of TMZ in melanoma patients [8]. They reported their retrospective experience with patients receiving this regimen alone or combined with either THA or low-dose interferon-alpha. Lymphopenia was not more common in patients treated with TMZ combined with THA, compared to TMZ alone. CD4+ lymphopenia was seen in 60% of patients, leading to opportunistic infections as well as other infections indicative of T-cell dysfunction. They concluded that Pneumocystis pneumonia prophylaxis should be considered for patients who develop sustained lymphopenia on TMZ. We can conclude the same from our study. We observed grade 2–4 lymphopenia during treatment in 27 of the 40 patients (67%). Interestingly, in this subgroup of patients significantly more responders were seen, indicating that immune parameters might influence the response of this combination regimen. From the study of Su *et al* [8], analysis by flow cytometry showed that the absolute CD4+ T-cell counts were depressed and that the CD4+CD25+ T-cell subset in some patients was markedly depressed, indicating that the lymphopenic effect of TMZ is relatively specific for the CD4+ subset.

Lately, it has become clear that T_reg_ cells might play a role in inhibiting beneficial anti-tumour immune responses [28]. Treatment of cancer patients with low doses of cyclophosphamide has resulted in the depletion of T_reg_ cells, which in theory might improve anti-tumour immunity [28,29]. Thus, we evaluated the impact of TMZ and THA on the T_reg_ cell populations to determine whether the treatment had any effect on these cells. When analysing the presence of CD4^+^CD25^+^FOXP3^+^ T_regs_ in patients treated with TMZ and THA, we found that the percentage of T_reg_ cells out of CD4 T cells was increased after treatment. However, this increase in T_reg_ cell proportion did not result in an increased total number of T_reg_ cells, since the percentage of CD4 T cells after treatment was lowered. Others have observed a preferential lymphopenia in the CD4^+^ compartment in melanoma patients after treatment with TMZ^8^. In the present study, we also observed a lowered percentage of CD4^+^ T cells out of total lymphocytes along with a lowered total number of CD4^+^ T cells after treatment with TMZ and THA. This indicates that other subsets of CD4^+^ T cells apart from the T_reg_ cell subset are sensitive to the treatment. Interestingly, when evaluating the fraction of CD4^+^CD127^+^CD25^neg-int^ out of CD4^+^ T cells, we found that this was lowered. Furthermore, we observed a tendency towards a lower number of total CD4^+^CD127^+^CD25^neg-int^ T cells after treatment, suggesting that these cells were influenced by TMZ. The expression of CD127 is down regulated on activated effector CD4 T cells [30], as well as on T_reg_ cells [31,32], thus it seems that TMZ affects the CD127^+^CD25^neg-int^ subset, which mostly contains conventional naïve T cells [33] and memory T cells [34]. Unfortunately, we only obtained data from patients with PD and SD. Thus, we are unable to report whether the CD4 T cell subsets fluctuate in a similar way in patients responding to the treatment with TMZ and THA or if they change at all. This will be evaluated in an ongoing trial.

The TMZ and THA treatment was well tolerated with mainly grade 1–2 non-haematological toxicity and no significant haematological toxicity. However, the combination had significantly more TEE than either Thalidomide or TMZ alone [18,35]. It has previously been shown in multiple myeloma that, when THA is combined with cytotoxic agents or with steroids, the frequency of TEE increases [36]. Furthermore, it is well known that cancer in itself is associated with a higher risk of TEE [37]. This was the case in the study performed by CALGB [24], which was suspended and subsequently closed because of severe TEE and limited number of objective responses. In the CALGB study, THA was given in doses up to 400 mg daily. The lower dose of THA in our study may explain the more manageable TEEs in four patients having deep-vein thrombosis and none of the patients developing pulmonary embolism. No significance can be established because of the small number of patients; however, in future studies one should be aware of this complication associated with this treatment combination and should maybe use lower doses of THA.

We conclude that the combination of TMZ, using the bi-weekly dose-intense schedule and THA 100–200 mg/d, is a safe regimen, leading to clinical efficacy in patients with brain metastases from malignant melanoma. Most importantly, it seems that patients who develop lymphopenia during therapy have a higher chance of obtaining objective response. The potential immunologic mechanism behind this will be the subject of future investigations, focusing on the potential benefit of T_reg_ down regulation. Further evaluations using larger patient numbers and including different therapy schedules will be considered for the future. Concerning the schedule of TMZ, we await the ongoing EORTC 18032 phase-III trial, investigating the bi-weekly regimen of TMZ versus standard Dacarbazine in previously untreated patients with metastatic melanoma.

## Figures and Tables

**Table 1:**
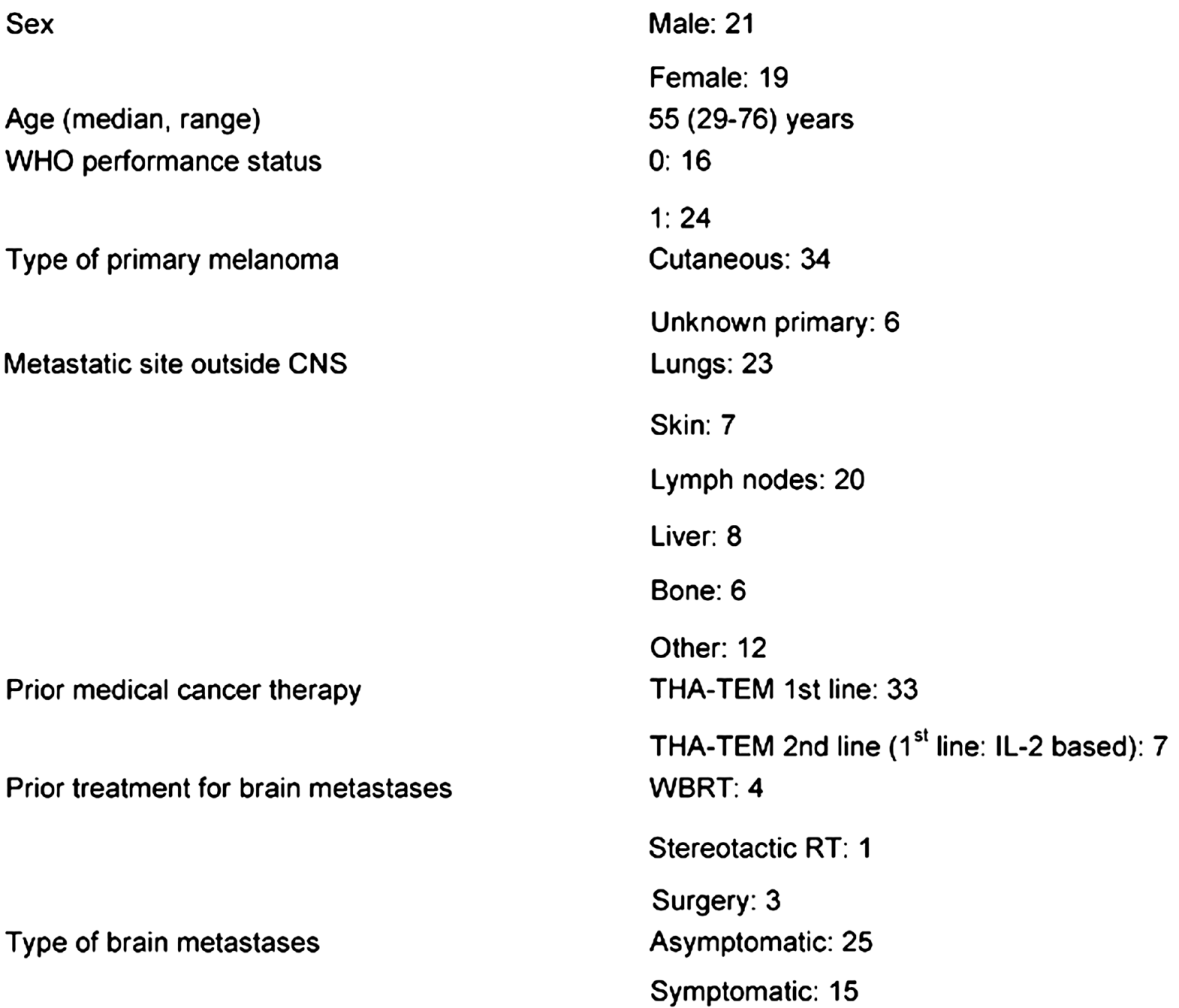
Patient characteristics (*n* = 40)

**Table 2:**
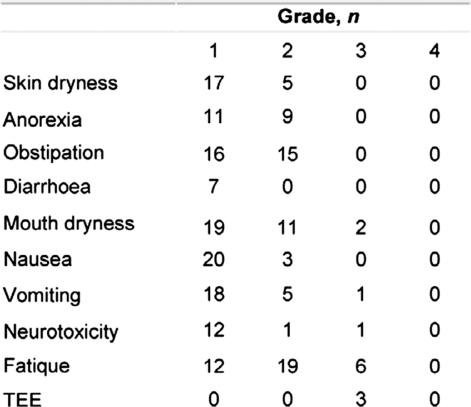
Toxicity profile in 39 evaluable patients

**Table 3:**
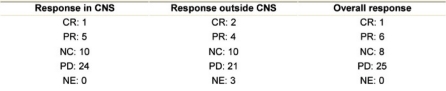
Response in 40 patients

**Table 4:**
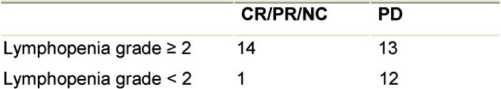
Significant correlation between response and lymphocyte count p2=0.01

## Supplementary figures

Figure 1: Percentage and total number of Treg cells in malignant melanoma patients before and after treatment with thalidomide and temozolomide. MNCs were stained with PE anti-human FOXP3, PerCP anti-human CD4, APC anti-human CD25. For defining the population of Treg cells, the cells were first gated on CD4+ lymphocytes then on CD4+ and CD25high. On the pre-sample, the level of CD4+CD25high out of CD4+ T cells was fixed at 2% and the following two samples (day 14 and day 28) were analysed on the same day using the same gates. To confirm that the cells within the gate were Treg cells, we evaluated the FOXP3 expression and found the FOXP3 expression to be between 80%–95%. (A) Percentage of Treg cells out of CD4 T cells. (B) Total number of Treg cells/μl blood

Figure 2: Percentage and total number of CD4 T cells in malignant melanoma patients before and after treatment with thalidomide and temozolomide. (A) Percentage of CD4 T cells out of lymphocytes. (B) Total number of CD4 T cells

Figure 3: Percentage and total number of CD4+CD25neg-intCD127+ T cells in malignant melanoma patients before and after treatment with thalidomide and temozolomide. MNCs were stained with FITC anti-human CD127, PerCP anti-human CD4, APC anti-human CD25. When evaluating the level of CD4+CD127+CD25neg-int T cells, the cells were gated on CD4+ lymphocytes then on CD127+ and CD25neg-int

## References

[1] Lee ML, Tomsu K, Von Eschen KB (2000). Duration of survival for disseminated malignant melanoma: results of a meta-analysis. Melanoma Res.

[2] Tarhini AA, Agarwala SS (2004). Management of brain metastases in patients with melanoma. Curr Opin Oncol.

[3] Meier S, Baumert BG, Maier T (2004). Survival and prognostic factors in patients with brain metastases from malignant melanoma. Onkologie.

[4] Sampson JH, Carter JH, Friedman AH (1998). Demographics, prognosis, and therapy in 702 patients with brain metastases from malignant melanoma. J Neurosurg.

[5] Payne MJ, Pratap SE, Middleton MR (2005). Temozolomide in the treatment of solid tumours: current results and rationale for dosing/scheduling. Crit Rev Oncol Hematol.

[6] Brock CS, Newlands ES, Wedge SR (1998). Phase I Trial of Temozolomide Using an Extended Continuous Oral Schedule. Cancer Res.

[7] Tolcher AW, Gerson SL, Denis L (2003). Marked inactivation of O6-alkylguanine-DNA alkyltransferase activity with protracted temozolomide schedules. Br J Cancer.

[8] Su YB, Sohn S, Krown SE, Livingston PO, Wolchok JD, Quinn C, Foster T, Sepkowitz KA, Chapman PB (2004). Selective CD4+ lymphopenia in melanoma patients treated with temozolomide: A toxicity with therapeutic implications. J Clin Oncol.

[9] Rietschel P, Wolchok JD, Krown S (2008). Phase II Study of Extended-Dose Temozolomide in Patients With Melanoma. J Clin Oncol.

[10] Thomas DA, Kantarjian HM (2000). Current role of thalidomide in cancer treatment. Curr Opin Oncology.

[11] D’Amato RJ, Loughnan MS, Flynn E (1994). Thalidomide is an Inhibitor of Angiogenesis. Proc Natl Acad Sci USA.

[12] Rajkumar SV (2001). Thalidomide in the treatment of plasma cell malignancies. J Clinical Oncol.

[13] Moreira AL, Sampaio EP, Zmuidzinas A (1993). Thalidomide exerts its inhibitory action on tumor necrosis factor alpha by enhancing mRNA degradation. J Exp Med.

[14] Meierhofer C, Wiedermann CJ (2003). New insights into the pharmacological and toxicological effects of Thalidomide. Curr Opin Drug Discov Devel.

[15] Meierhofer C, Dunzendorfer S, Wiedermann CJ (2001). Theoretical Basis for the Activity of Thalidomide. BioDrugs.

[16] Franks ME, MacPherson GR, Figg WD (2004). Thalidomide. Lancet.

[17] Kumar S, Witzig TE, Rajkumar SV (2004). Thalidomide: Current role in the treatment of non-plasma cell malignancies. J Clin Oncol.

[18] Vestermark LW, Larsen SU, Lindeløv B, Bastholt L (2008). A phase II study of Thalidomide in patients with brain metastases from malignant melanoma. Acta Oncologica.

[19] Hwu WJ, Lis E, Menell JH (2005). Temozolomide Plus Thalidomide in Patients with Brain Metastases from Melanoma. Cancer.

[20] Zeldis JB, Williams BA, Thomas SD (1999). STEPS: a comprehensive program for controlling and monitoring access to thalidomide. Clin Ther.

[21] Trotti A, Colevas AD, Setser A (2003). CTCAE v3.0: development of a comprehensive grading system for the adverse effects of cancer treatment. Seminars in Rad Oncol.

[22] Therasse P, Arbuck SG, Eisenhauer EA (2000). New guidelines to evaluate the response to treatment in solid tumors. J Natl Cancer Inst.

[23] Simon R (1989). Optimal two-stage designs for phase II clinical trials. Control Clin Trials.

[24] Krown SE, Niedzwiecki D, Hwu W-J (2006). Phase II study of temozolomide and thalidomide in patients with metastatic melanoma in the brain : High rate of thromboembolic events (CALGB 500102). Cancer.

[25] Boogerd W, de Gast GC, Dalesio O (2006). Temozolomide in advanced Malignant Melanoma with smal brain metastases. Can We Withhold cranial irradiation?. Cancer.

[26] Schadendorf D, Hauschild A, Ugurel S (2006). Dose-intensified bi-weekly temozolomide in patients with asymptomatic brain metastases from malignant melanoma: a phase II DeCOG/ADO study. Ann Oncol.

[27] Laber D, Okeke R, Arce-Lara C (2006). A phase II study of extended dose temozolomide and thalidomide in previously treated patients with metastatic melanoma. J Cancer Res Clin Oncol.

[28] Zou W (2006). Regulatory T cells, tumour immunity and immunotherapy. Nat Rev Immunol.

[29] Ghiringhelli F, Menard C, Puig P (2007). Metronomic cyclophosphamide regimen selectively depletes CD4+CD25+ regulatory T cells and restores T and NK effector functions in end stage cancer patients. Cancer Immunol Immunother.

[30] Lim HW, Kim CH (2007). Loss of IL-7 Receptor {alpha} on CD4+ T Cells Defines Terminally Differentiated B Cell-Helping Effector T Cells in a B Cell-Rich Lymphoid Tissue. J Immunol.

[31] Seddiki N, Santner-Nanan B, Martinson J (2006). Expression of interleukin (IL)-2 and IL-7 receptors discriminates between human regulatory and activated T cells. J Exp Med.

[32] Liu W, Putnam AL, Xu-yu Z (2006). CD127 expression inversely correlates with FoxP3 and suppressive function of human CD4+ T reg cells. J Exp Med.

[33] Fry TJ, Mackall CL (2005). The Many Faces of IL-7: From Lymphopoiesis to Peripheral T Cell Maintenance. J Immunol.

[34] Kondrack RM, Harbertson J, Tan JT (2003). Interleukin 7 Regulates the Survival and Generation of Memory CD4 Cells. J Exp Med.

[35] Agarwala SS, Kirkwood JM, Gore M (2004). Temozolomide for the Treatment of Brain Metastases Associated With Metastatic Melanoma: A Phase II Study. J Clin Oncol.

[36] Zangari M, Anaissie E, Barlogie B (2001). Increased risk of deep-vein thrombosis in patients with multiple myeloma receiving thalidomide and chemotherapy. Blood.

[37] Gerber DE, Grossman SA, Streiff MB (2006). Management of Venous Thromboembolism in Patients With Primary and Metastatic Brain Tumors. J Clin Oncol.

