# Supplementary figures and images for: A phase II study of thalidomide and temozolomide in patients with brain metastases from malignant melanoma: lymphopenia correlates with response
Source: Ecancermedicalscience. 2008 Aug 15;2:91. doi: 10.3332/ecancer.2008.91 (PMC3234039; doi:10.3332/ecancer.2008.91)

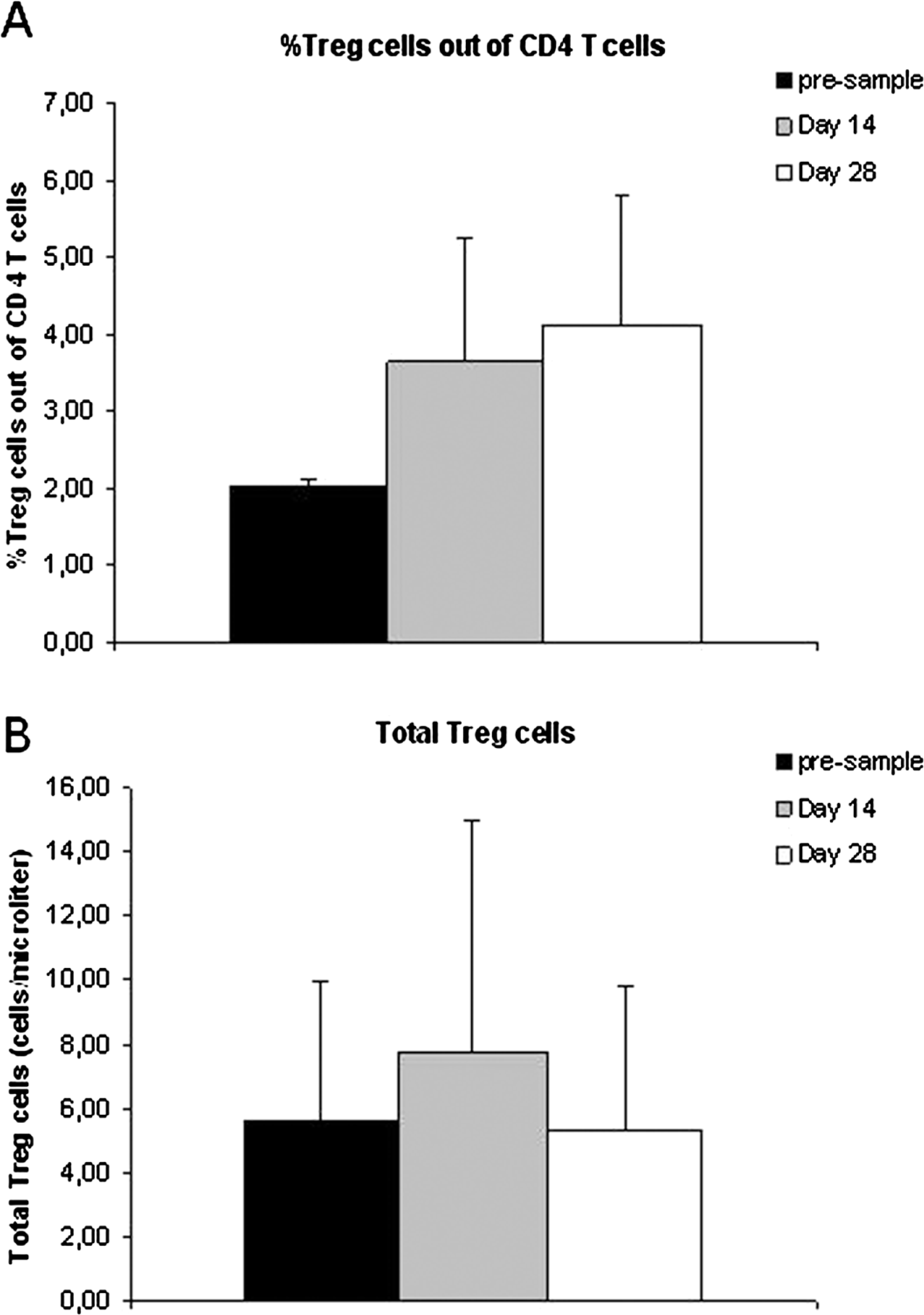

Supplement: Figure 1: — Percentage and total number of Treg cells in malignant melanoma patients before and after treatment with thalidomide and temozolomide. MNCs were stained with PE anti-human FOXP3, PerCP anti-human CD4, APC anti-human CD25. For defining the population of Treg cells, the cells were first gated on CD4+ lymphocytes then on CD4+ and CD25high. On the pre-sample, the level of CD4+CD25high out of CD4+ T cells was fixed at 2% and the following two samples (day 14 and day 28) were analysed on the same day using the same gates. To confirm that the cells within the gate were Treg cells, we evaluated the FOXP3 expression and found the FOXP3 expression to be between 80%–95%. (A) Percentage of Treg cells out of CD4 T cells. (B) Total number of Treg cells/μl blood [file can-2-91s1.tif]

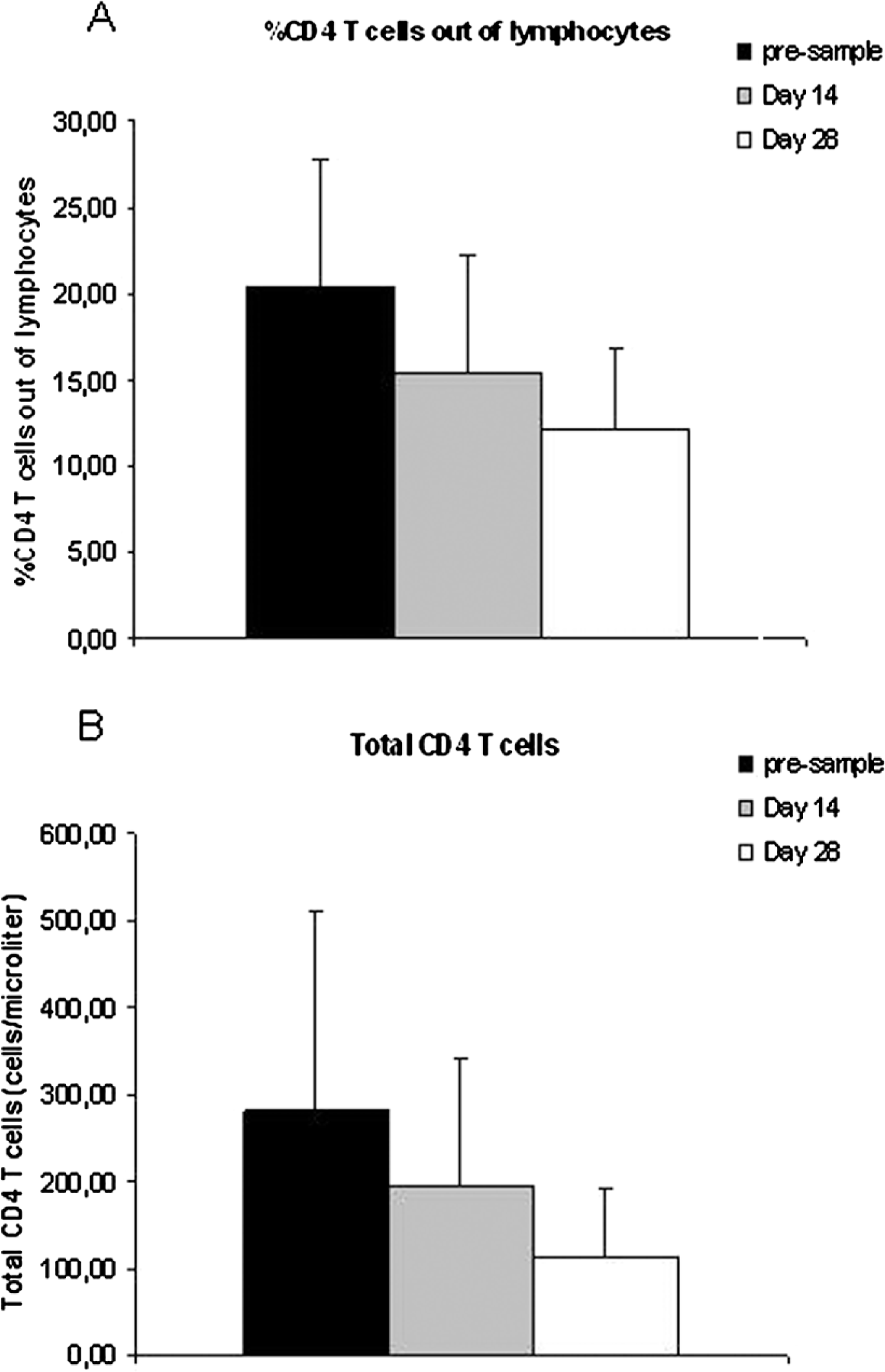

Supplement: Figure 2: — Percentage and total number of CD4 T cells in malignant melanoma patients before and after treatment with thalidomide and temozolomide. (A) Percentage of CD4 T cells out of lymphocytes. (B) Total number of CD4 T cells [file can-2-91s2.tif]

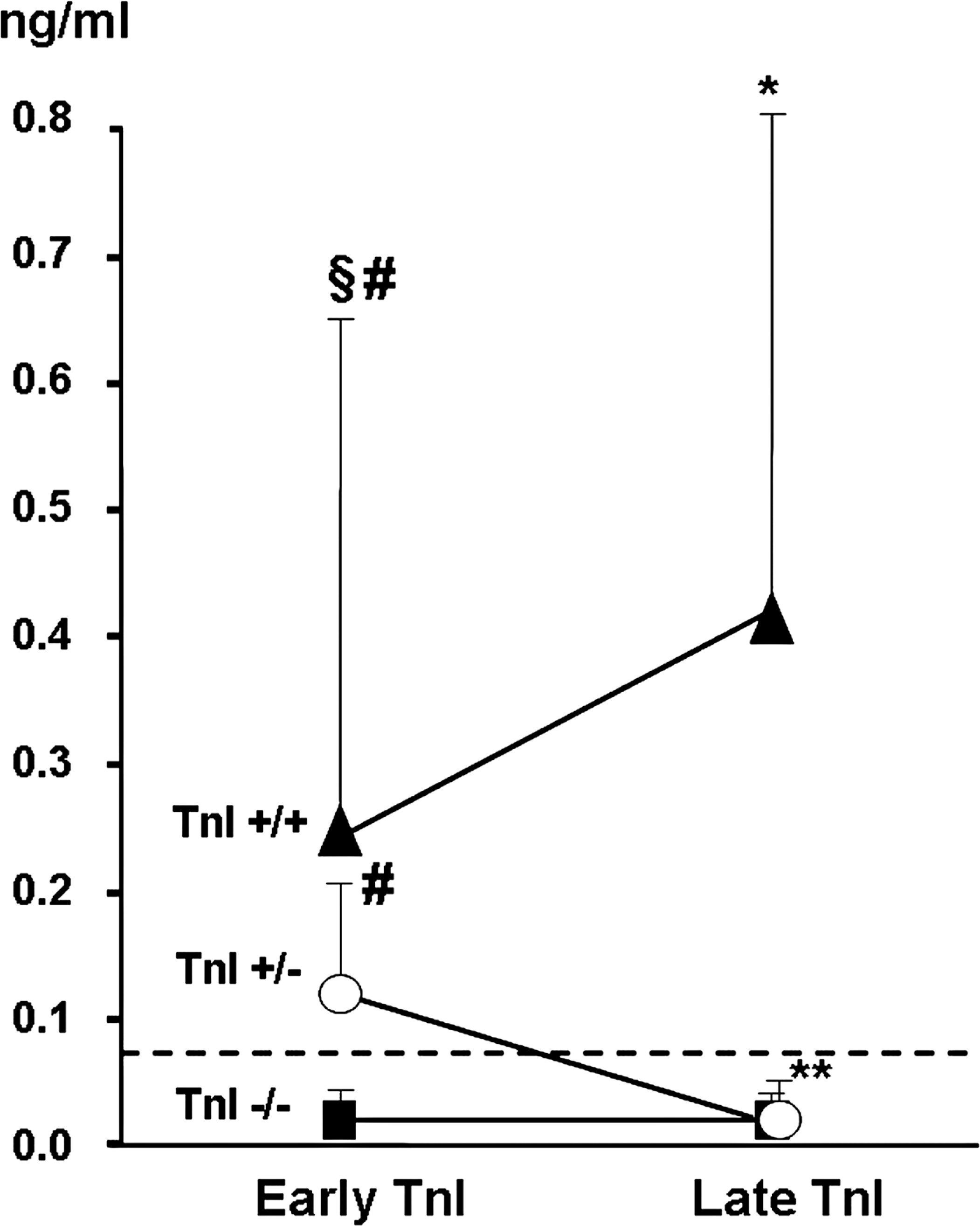

Supplement: Figure 3: — Percentage and total number of CD4+CD25neg-intCD127+ T cells in malignant melanoma patients before and after treatment with thalidomide and temozolomide. MNCs were stained with FITC anti-human CD127, PerCP anti-human CD4, APC anti-human CD25. When evaluating the level of CD4+CD127+CD25neg-int T cells, the cells were gated on CD4+ lymphocytes then on CD127+ and CD25neg-int [file can-2-91s3.tif]
